# Hypovitaminosis D and Insulin Resistance in Type 1 Diabetes Mellitus: A Systematic Review of Clinical and Mechanistic Evidence

**DOI:** 10.7759/cureus.106061

**Published:** 2026-03-29

**Authors:** Yasir Ahmed, Ali Hadi M Alhajri, Sami M Osman, Wadah Ahmed Osman Ahmed, Sawsan Musa Esmail Musa, Rawan Ahmed, Rayan Saad Aldeen Mohammed Saad Aldeen

**Affiliations:** 1 Acute Medicine, University Hospital of North Midlands, Stoke-On-Trent, GBR; 2 Endocrinology, Najran Armed Forces Hospital, Ministry of Defense Health Services, Najran, SAU; 3 School of Medicine, St. George's University, St. George's, GRD; 4 Internal Medicine, Kalba Hospital, Sharjah, ARE; 5 Endocrinology, Almahani Hospital, Taif, SAU; 6 General Practice, Glenfield Hospital, Leicester, GBR; 7 Internal Medicine, Igraa College for Science and Technology, Wad Madani, SDN

**Keywords:** 25-hydroxyvitamin d, insulin resistance, systematic review, type 1 diabetes mellitus, vitamin d deficiency

## Abstract

Type 1 diabetes mellitus (T1DM) involves autoimmune β-cell destruction, but insulin resistance may also influence disease outcomes. Vitamin D modulates insulin sensitivity and immune function, and hypovitaminosis D is common in T1DM. This systematic review evaluates clinical and mechanistic evidence on the association between hypovitaminosis D and insulin resistance in T1DM.

A Preferred Reporting Items for Systematic Reviews and Meta‑Analyses (PRISMA) framework was applied for a systematic search of PubMed^®^, BMJ Journals, Scopus^®^, IEEE Xplore^®^, and Web of Science™, including articles published until 7 March 2026. Studies assessing vitamin D status and insulin resistance measures in T1DM populations were included. Risk of bias was evaluated using the Cochrane Risk of Bias 2 tool and the Newcastle-Ottawa Scale. Narrative synthesis was performed due to methodological heterogeneity. Eight studies (one controlled trial, two prospective cohorts, and five cross-sectional) were included. Hypovitaminosis D prevalence ranged from 47 to 79%. Six studies reported significant associations between low vitamin D levels and markers of insulin resistance, including a positive correlation with estimated glucose disposal rate (eGDR), as well as associations with higher insulin requirements and greater odds of insulin resistance. Mechanistic studies demonstrated preserved β-cell function with sufficient vitamin D and identified vitamin D receptor (VDR) polymorphisms as effect modifiers. Supplementation trials showed conflicting results, and longitudinal analysis revealed no significant association over time. Risk of bias was low in one study, good in five, and fair in two. Hypovitaminosis D is prevalent in T1DM and associated with insulin resistance in cross-sectional studies, with supportive mechanistic evidence. However, interventional and longitudinal data remain inconsistent. Vitamin D may be a marker of metabolic dysregulation, but its therapeutic role in improving insulin resistance requires further robust investigation.

## Introduction and background

Type 1 diabetes mellitus (T1DM) is a chronic autoimmune disorder characterised by immune-mediated destruction of pancreatic β-cells, resulting in absolute insulin deficiency and lifelong dependence on exogenous insulin therapy [1]. While T1DM has traditionally been viewed as a condition driven primarily by insulin deficiency, emerging evidence indicates that insulin resistance also contributes to its clinical course and metabolic complications, affecting an estimated 20-50% of individuals with the disease [2]. The presence of insulin resistance in T1DM has been associated with poor glycaemic control, increased insulin requirements, and a higher risk of both microvascular and macrovascular complications, underscoring the importance of identifying modifiable factors influencing insulin sensitivity in this population [3].

Vitamin D, a fat-soluble secosteroid hormone best known for its role in calcium homeostasis and bone metabolism, has also been implicated in a range of metabolic and immunological processes [4]. Its biological effects are mediated through the vitamin D receptor (VDR), which is expressed in key tissues involved in glucose metabolism, including pancreatic β-cells, skeletal muscle, adipose tissue, and immune cells [5]. Through these pathways, vitamin D may influence insulin secretion, modulate inflammatory responses, and enhance insulin sensitivity, suggesting a potential role in glucose homeostasis beyond skeletal health [6].

Globally, vitamin D deficiency is common and has been frequently reported in individuals with T1DM [7]. Despite growing interest, studies examining the relationship between vitamin D status and insulin resistance in T1DM have yielded inconsistent findings, likely due to heterogeneity in study populations, methodologies, and outcome measures. Additionally, limited consideration has been given to population diversity and contextual factors that may influence both vitamin D status and metabolic outcomes. Therefore, a comprehensive synthesis of the available clinical and mechanistic evidence is warranted. This systematic review aims to critically evaluate the association between hypovitaminosis D and insulin resistance in individuals with T1DM.

## Review

Methodology

Study Design

This systematic review was conducted in accordance with the recommendations of the Preferred Reporting Items for Systematic Reviews and Meta‑Analyses (PRISMA) guidelines [8] to ensure transparency, methodological rigour, and reproducibility. All stages of the review, including literature searching, study selection, data extraction, and risk of bias assessment, were conducted using predefined criteria to minimise selection bias and improve methodological consistency. PROSPERO registration was not performed, as the review includes mechanistic and observational studies beyond interventional trials, which fall outside PROSPERO's typical scope.

Information Sources

A comprehensive literature search was performed across multiple electronic databases to identify relevant studies. The databases searched included PubMed^®^, BMJ Journals, Scopus^®^, IEEE Xplore^®^, and Web of Science™. These databases were selected because they provide extensive coverage of biomedical, clinical, and interdisciplinary research relevant to diabetes, endocrinology, and metabolic studies. The search strategy was designed to capture studies investigating vitamin D status and insulin resistance in individuals with T1DM. To ensure comprehensive coverage of the available evidence, no restrictions were placed on the publication year, allowing inclusion of all relevant studies from database inception to the date of the final search, which was 7 March 2026.

Search Strategy

A structured search strategy was developed using a combination of Medical Subject Headings (MeSH) and relevant keywords related to vitamin D deficiency, insulin resistance, and type 1 diabetes mellitus. The search terms included combinations of keywords such as "vitamin D deficiency", "hypovitaminosis D", "25-hydroxyvitamin D", "type 1 diabetes mellitus", and "insulin resistance". Boolean operators (AND, OR) were applied to optimise the search sensitivity and specificity. The search strategy was adapted appropriately for each database to account for differences in indexing systems and search functionalities. Additionally, the reference lists of eligible studies were manually screened to identify any potentially relevant articles that may have been missed during the database search. The full search strategy for each database is provided in the appendix.

Eligibility Criteria

The eligibility criteria for study inclusion were defined according to the population, intervention, comparison, outcome, and study design (PICOS) framework. The population (P) included individuals diagnosed with type 1 diabetes mellitus, regardless of age, gender, or geographic location. The intervention/exposure (I) consisted of studies evaluating vitamin D status, particularly hypovitaminosis D or serum 25-hydroxyvitamin D levels. The comparison (C) included individuals with T1DM who had adequate or sufficient vitamin D levels, or, when available, comparative groups within the same T1DM population. The outcomes (O) of interest were measures of insulin resistance or insulin sensitivity, including indices such as the Homeostatic Model Assessment of Insulin Resistance (HOMA-IR), estimated glucose disposal rate (eGDR), insulin dose requirements, or other validated metabolic markers related to insulin resistance. The study design (S) included original research studies such as observational studies (cross-sectional, case-control, and cohort studies), clinical trials, and mechanistic or experimental investigations examining the relationship between vitamin D status and insulin resistance in T1DM. Review articles, editorials, conference abstracts without full text, animal-only studies without human relevance, and studies not reporting outcomes related to insulin resistance were excluded.

Study Selection

All records retrieved from the electronic databases were exported into EndNote™ X9 (Clarivate, London, UK) reference management software for organisation and screening. Duplicate records were identified and removed automatically and manually using the duplicate detection functions within the software. After duplicate removal, the remaining titles and abstracts were screened to identify potentially relevant studies. Full texts of studies meeting the preliminary eligibility criteria were then retrieved and assessed for inclusion according to the predefined PICOS criteria. Studies that did not meet the inclusion criteria after full-text review were excluded, with reasons documented to ensure transparency in the selection process.

Data Extraction

Relevant data from the included studies were systematically extracted using a standardised data extraction approach to ensure consistency across studies. Extracted information included study characteristics such as author name, year of publication, country, study design, sample size, participant demographics, methods used to measure vitamin D levels, definitions of hypovitaminosis D, and the methods used to assess insulin resistance. Additional information regarding statistical associations, mechanistic findings, and key conclusions reported by the authors was also recorded. This structured extraction allowed for a comprehensive comparison of clinical and mechanistic evidence across the included studies.

Risk of Bias Assessment

The methodological quality and risk of bias of the included studies were evaluated using validated assessment tools appropriate for different study designs. Randomised controlled trials were assessed using the Cochrane Risk of Bias Tool 2 (RoB 2) [9], while observational studies were evaluated using the Newcastle-Ottawa Scale (NOS) [10]. These tools assess potential bias across several domains, including selection bias, performance bias, detection bias, and reporting bias. Each included study was carefully evaluated to determine its methodological robustness and the reliability of its findings. The results of the risk of bias assessment were considered when interpreting the overall evidence.

Data Synthesis

A qualitative narrative synthesis was performed to summarise the findings of the included studies. Although several studies examined the association between vitamin D status and insulin resistance in T1DM, a quantitative meta-analysis was not conducted. The primary reason for this decision was the substantial methodological heterogeneity across the included studies, including differences in study designs, populations, definitions of vitamin D deficiency, methods used to measure serum vitamin D levels, and various indices employed to assess insulin resistance. Additionally, the variability in reported outcomes and statistical measures limited the ability to generate statistically comparable effect sizes across studies. Conducting a meta-analysis under such conditions could produce misleading pooled estimates and compromise the validity of the findings. Therefore, a qualitative synthesis was considered the most appropriate approach to accurately integrate and interpret the available clinical and mechanistic evidence.

Results

Study Selection Process

**Figure 1:**
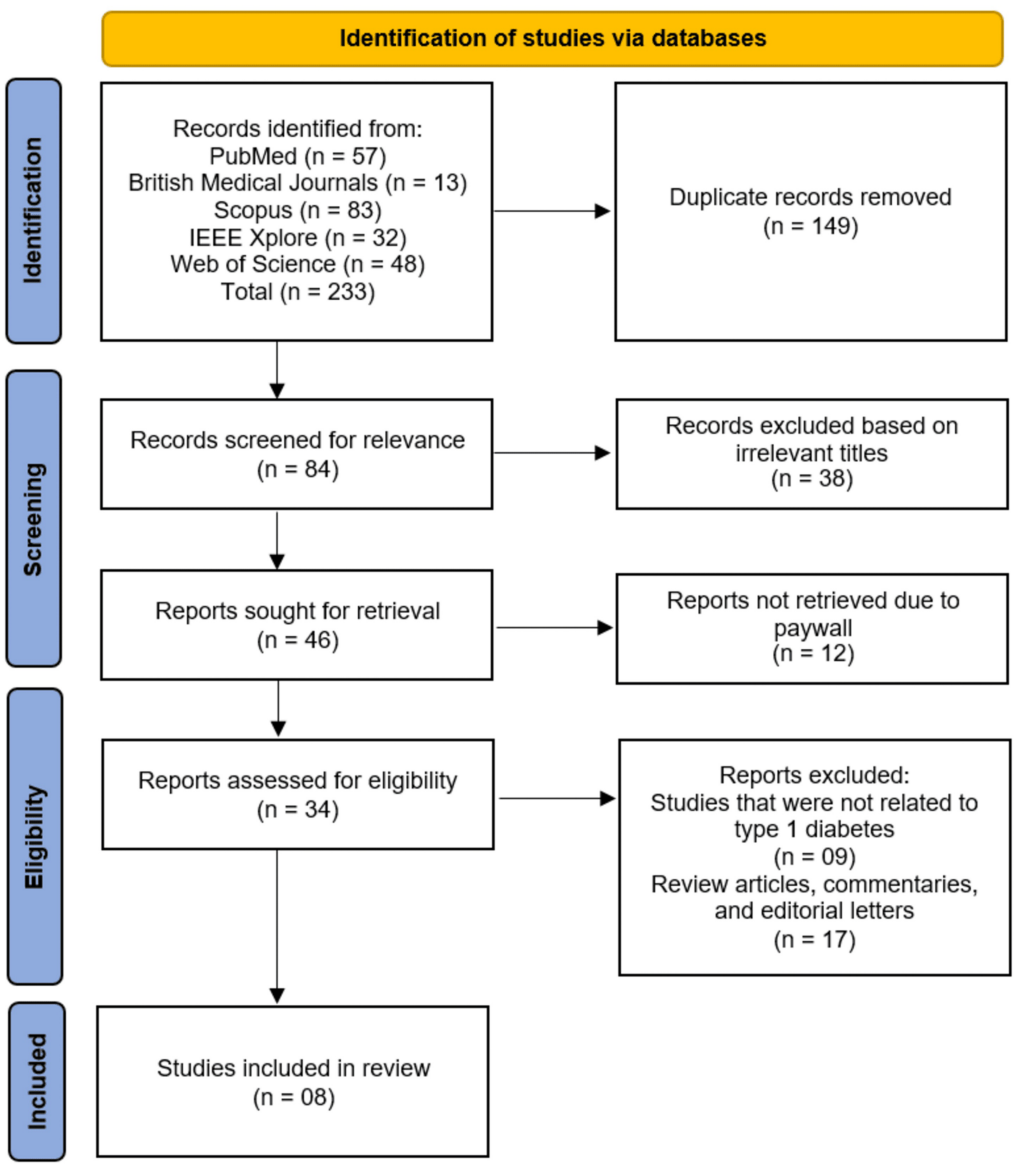
Study selection process on the PRISMA flowchart PRISMA - Preferred Reporting Items for Systematic Reviews and Meta‑Analyses

The systematic literature search identified a total of 233 records from five electronic databases, including PubMed^®^ (n=57), British Medical Journals (n=13), Scopus® (n=83), IEEE Xplore^®^ (n=32), and Web of Science™ (n=48). After the removal of 149 duplicate records, 84 studies remained for initial screening based on relevance. Following the screening of titles and abstracts, 38 records were excluded due to irrelevant titles, leaving 46 reports sought for retrieval. Of these, 12 reports could not be retrieved due to paywall restrictions. The remaining 34 reports were assessed for eligibility through full-text review. During this eligibility phase, no studies were excluded because they were not related to type 1 diabetes mellitus, and a further 17 reports were excluded as they were review articles, commentaries, or editorial letters. Following this rigorous selection process, a total of eight studies [11-18] met the inclusion criteria and were included in this systematic review. The study selection process is illustrated in the PRISMA flow diagram (Figure 1).

Characteristics of Included Studies

The characteristics of these studies are summarised in Table 1. The included studies comprised one controlled clinical trial [11], two prospective cohort studies [16,17], one retrospective cross-sectional study [15], three cross-sectional studies [12-14], and one study with both cross-sectional and longitudinal components [18]. Geographically, the studies were conducted across diverse regions, including Brazil [11], Poland [12], Turkey [13], Italy [14], Saudi Arabia [15], Finland [16], Iran [17], and the United States [18]. The study populations varied in age range, with six studies focusing on paediatric and adolescent populations [11,13-17], one study including both children and adults [18], and one study specifically recruiting adults with type 1 diabetes [12]. Sample sizes ranged from 100 to 1426 participants across the included studies.

**Table 1:** Characteristics of included studies T1DM - type 1 diabetes mellitus; 25(OH)D - 25-hydroxyvitamin D; VDR - vitamin D receptor; IR - insulin resistance; CLIA - chemiluminescence immunoassay; ELISA - enzyme-linked immunosorbent assay; eGDR - estimated glucose disposal rate; HPLC - high-performance liquid chromatography

Author (year)	Country	Study design	Population (sample size)	Age range/ mean age	Vitamin D measurement	Definition of hypovitaminosis D	Insulin resistance assessment	Key objective
Moreira et al., [11] (2025)	Brazil	Controlled clinical trial	T1DM children/adolescents (n=133)	7-16 years; mean 11.4	Serum 25(OH)D (CLIA)	25(OH)D <30 ng/mL	HbA1c	Assess the effect of vitamin D supplementation on glycaemic control in T1DM
Kamiński et al., [12] (2021)	Poland	Cross-sectional	T1DM adults (n=110)	18-50	Serum 25(OH)D (ELISA)	Deficiency <20 ng/mL, severe <10	eGDR <7.5 mg/kg/min	Association of vitamin D with IR
Tunc et al., [13] (2011)	Turkey	Cross-sectional	T1DM children (n=100)	4.7-19.9/12.5	25(OH)D (HPLC)	<10 ng/mL def, 10-20 insuff	Insulin U/kg	Link vitamin D and insulin
Savastio et al., [14] (2016)	Italy	Cross-sectional	n=141	13.3±4.3	25(OH)D chemiluminescence	<75/50/25 nmol/L	Insulin dose, HbA1c	25(OH)D status and glycaemic control; effect of supplementation
Al Zahrani and Al Shaikh, [15] (2019)	Saudi Arabia	Retrospective cross-sectional	Children and adolescents with T1DM (n=301)	1-18 years; 13.9±3.8	25(OH)D	Not explicitly defined; mean 25(OH)D 35.1±15.9 nmol/L	HbA1c (glycaemic control)	Assess vitamin D status and its association with glycaemic control in T1DM children
Mäkinen et al., [16] (2016)	Finland	Prospective cohort (nested case-control)	Children (T1DM cases n=126, controls n=126; total n=252 )	Median ~2.9 years; 0.15-14.23	Serum 25(OH)D immunoassay	Severe deficiency <25 nmol/L; 50-75 nmol/L inadequate	Not assessed	Assess association of 25(OH)D with T1DM development
Habibian et al., [17] (2019)	Iran	Prospective cohort	T1DM patients (Persian ethnicity, n=101)	5-15 years; mean 9.28±2.54	Chemiluminescent method (ARCHITECT kit)	Deficient <10 ng/ml; Insufficient 10-30 ng/ml; sufficient >30 ng/ml	Fasting and stimulated C-peptide levels	Assess the relationship between vitamin D levels, VDR polymorphisms, and β-cell function in newly diagnosed T1DM children
The et al., [18] (2013)	USA	Cohort (cross-sectional and longitudinal)	T1DM, cross-sectional (n=1426); longitudinal (n=735)	≥10 years; 10.3±3.6 years (longitudinal)	25(OH)D, chemiluminescence	<30 nmol/L (deficiency), 30-49.9 nmol/L (inadequacy)	Insulin sensitivity score; IR<8.15	Association of vitamin D with IR

Vitamin D Status in Type 1 Diabetes Populations

The prevalence of hypovitaminosis D among individuals with type 1 diabetes varied considerably across studies, partly due to different threshold definitions for deficiency and insufficiency. Moreira and colleagues reported that 79% of children and adolescents with type 1 diabetes had vitamin D deficiency, defined as serum 25-hydroxyvitamin D (25(OH)D) below 30 ng/mL [11]. In their controlled clinical trial, baseline 25(OH)D levels increased significantly from 19.2 to 30.9 ng/mL (p<0.001) following supplementation [11]. Kamiński et al. found that 47.3% of adults with type 1 diabetes were vitamin D deficient (<20 ng/mL), with 14.5% exhibiting severe deficiency (<10 ng/mL) [12]. Similarly, Tunc and colleagues observed varying degrees of hypovitaminosis in their paediatric cohort, classifying participants as deficient or insufficient based on 25(OH)D thresholds [13]. Savastio et al. reported that most of their paediatric participants were either insufficient or deficient in vitamin D, and supplementation effectively improved 25(OH)D levels [14]. Al Zahrani and Al Shaikh documented a mean 25(OH)D concentration of 35.1±15.9 nmol/L in their Saudi Arabian cohort, indicating a high prevalence of deficiency, although a specific threshold for deficiency was not explicitly defined [15]. Mäkinen and colleagues, in their nested case-control study, reported median 25(OH)D levels of 66.6 nmol/L in children who developed type 1 diabetes compared to 67.4 nmol/L in controls, with most participants falling within the insufficient range [16]. Habibian et al. categorised their participants as deficient (<10 ng/mL), insufficient (10-30 ng/mL), or sufficient (>30 ng/mL), finding considerable variation in vitamin D status among newly diagnosed children [17]. The et al. reported that in their large cohort of youth with type 1 diabetes, deficiency (<30 nmol/L) and inadequacy (30-49.9 nmol/L) were common when compared to sufficient status (≥50 nmol/L) [18].

Association Between Vitamin D and Insulin Resistance Measures

The relationship between vitamin D status and insulin resistance in type 1 diabetes was assessed using various outcome measures, including estimated glucose disposal rate (eGDR), insulin dose requirements, HbA1c, and insulin sensitivity scores. Kamiński and colleagues demonstrated a positive correlation between 25(OH)D levels and eGDR (Rs=0.27, p<0.01), with severe vitamin D deficiency (<10 ng/mL) conferring a substantially increased odds ratio for insulin resistance (OR=4.19) [12]. Tunc et al. found a significant negative correlation between 25(OH)D and insulin requirements (r=-0.215, p=0.032), with adequately vitamin D replete patients having significantly lower insulin needs compared to those with deficiency or insufficiency (p=0.012) [13]. Savastio and colleagues reported negative correlations between vitamin D status and both HbA1c and insulin dose, with significant improvements observed following vitamin D supplementation, suggesting enhanced insulin sensitivity [14]. In contrast, Al Zahrani and Al Shaikh found no association between vitamin D levels and HbA1c (R=0.04, p=0.60) in their cross-sectional analysis of Saudi children and adolescents [15]. The et al., utilising data from the SEARCH Nutrition Ancillary Study, demonstrated a significant cross-sectional association between higher vitamin D levels and lower insulin resistance, with an odds ratio of 0.70 (95% CI: 0.57-0.85) for insulin resistance in those with sufficient versus insufficient vitamin D [18]. However, their longitudinal analysis did not show a statistically significant association (OR: 0.69, 95% CI: 0.63-1.14) [18]. Moreira and colleagues, using HbA1c as a proxy for insulin resistance, found no significant change in HbA1c following 12 weeks of vitamin D supplementation (p=0.171), with a very small effect size observed [11].

Mechanistic Findings and Beta-Cell Function

Several studies investigated potential mechanisms underlying the relationship between vitamin D and insulin resistance in type 1 diabetes. Habibian and colleagues provided mechanistic insights by demonstrating that children with sufficient vitamin D levels had significantly higher stimulated C-peptide levels compared to those with deficiency (p<0.01), suggesting that vitamin D may protect residual beta-cell function through anti-apoptotic and anti-inflammatory mechanisms. They also reported that vitamin D receptor (VDR) gene polymorphisms modulated this effect, indicating a genetic component to vitamin D's influence on beta-cell preservation [17]. Savastio et al. proposed that hypovitaminosis D worsens metabolic control through both reduced insulin sensitivity and potential direct effects on beta-cell and peripheral tissue function [14]. Tunc and colleagues observed that low 25(OH)D levels were associated with elevated parathyroid hormone and reduced insulin sensitivity, suggesting a potential endocrine mechanism linking vitamin D status to insulin action [13]. Kamiński et al. found that low vitamin D levels were associated with higher waist-to-hip ratio and systolic blood pressure, which may contribute to insulin resistance through metabolic syndrome pathways [12]. Mäkinen and colleagues, while not directly assessing insulin resistance, noted seasonal variation in vitamin D levels and an inverse association with BMI, though they found no significant difference in 25(OH)D concentrations between children who progressed to type 1 diabetes and controls (p=0.56) [16]. The et al. reported that lower vitamin D status was associated with higher BMI, elevated triglycerides, and increased HbA1c, suggesting that vitamin D may influence insulin resistance through multiple metabolic pathways [18]. Moreira and colleagues examined the Fok-I VDR polymorphism but found no association with HbA1c, though they noted that sedentary lifestyle and higher BMI tended to correlate with lower vitamin D levels [11].

Effects of Vitamin D Supplementation

Two studies provided data on the effects of vitamin D supplementation on metabolic outcomes in type 1 diabetes. Moreira and colleagues, in their controlled clinical trial, demonstrated that vitamin D supplementation effectively corrected deficiency, increasing 25(OH)D from 19.2 to 30.9 ng/mL (p<0.001), but this did not translate into significant improvement in glycaemic control as measured by HbA1c [11]. In contrast, Savastio and colleagues reported that supplementation improved 25(OH)D status and was associated with enhanced insulin sensitivity and better glycaemic outcomes, supporting a beneficial role for vitamin D repletion in metabolic control [14]. These conflicting findings may reflect differences in study design, participant characteristics, duration of supplementation, or baseline vitamin D status.

Summary of Evidence

The collective evidence from the included studies, as summarised in Table 2, presents a mixed picture regarding the association between hypovitaminosis D and insulin resistance in type 1 diabetes. The majority of cross-sectional studies demonstrated significant associations between low vitamin D status and markers of insulin resistance, including higher insulin requirements, lower eGDR, and reduced insulin sensitivity [12-14,18]. Mechanistic studies suggested that vitamin D may influence insulin resistance through effects on beta-cell function, inflammatory pathways, and metabolic parameters [14,17]. However, intervention trials showed inconsistent results, with one study failing to demonstrate improved glycaemic control following supplementation [11], while another reported beneficial effects [14]. Longitudinal evidence was limited, with one large study showing no significant association over time despite cross-sectional findings [18]. The variability in findings may be attributable to differences in study populations, vitamin D assessment methods, threshold definitions for deficiency, and outcome measures employed across studies.

**Table 2:** Summary of clinical and mechanistic findings T1DM - type 1 diabetes mellitus; 25(OH)D - 25-hydroxyvitamin D; VDR - vitamin D receptor; eGDR - estimated glucose disposal rate; WHR - waist-to-hip ratio; SBP - systolic blood pressure; PTH - parathyroid hormone; SCP - serum C-peptide; FCP - fasting C-peptide; IR - insulin resistance

Author (year)	Vitamin D status in T1DM	Insulin resistance outcome	Statistical association	Mechanistic findings	Authors' conclusion
Moreira et al., [11] (2025)	79% of children with T1DM had vitamin D deficiency (<30 ng/mL); supplementation increased 25(OH)D from 19.2 to 30.9 ng/mL (p<0.001)	HbA1c used as a proxy for insulin resistance; levels remained largely unchanged after supplementation	No significant change in HbA1c after 12 weeks (p=0.171); very small effect size	Fok-I VDR polymorphism not associated with HbA1c; sedentary lifestyle and higher BMI tended to correlate with lower vitamin D levels	Vitamin D supplementation corrects deficiency but does not significantly improve glycaemic control in paediatric T1DM
Kamiński et al., [12] (2021)	Deficient (<20 ng/ml) 47.3%; severe <10 ng/ml 14.5%	eGDR <7.5 mg/kg/min; 19% IR	Positive correlation with eGDR (Rs=0.27, p<0.01); OR=0.95; severe deficiency OR=4.19	Low vitamin D linked to higher WHR and SBP	Low 25(OH)D associated with IR; severe deficiency is a strong risk factor
Tunc et al., [13] (2011)	Low (deficiency/insufficiency) vs adequate	Higher insulin requirement, lower sensitivity	Negative correrlation between 25(OH)D and insulin (r=-0.215, p=0.032); vs adequate (p=0.012)	Low 25(OH)D increases PTH and reduces insulin sensitivity	Low vitamin D may increase insulin requirements due to poorer glycaemic control; replacement improves control
Savastio et al., [14] (2016)	Mostly insufficient/deficient; supplementation improved 25(OH)D	Higher HbA1c and insulin dose with deficiency; lower with supplementation	Negative correlation with HbA1c and insulin dose; significant improvements with supplementation	Low insulin sensitivity, possible β-cell and peripheral effects; supports glycaemic control	Hypovitaminosis D worsens metabolic control in T1DM; supplementation improves insulin sensitivity and glycaemic outcomes
Al Zahrani and Al Shaikh, [15] (2019)	Mean 35.1±15.9 nmol/L; deficiency common	HbA1c 9.67±1.93%; 26% met target	No association (R=0.04, p=0.60)	Not directly studied	Vitamin D deficiency high; supplementation recommended; no effect on glycaemic control observed
Mäkinen et al., [16] (2016)	Similar median 25(OH)D levels in cases (66.6 nmol/L) and controls (67.4 nmol/L); mostly insufficient range	Not assessed	No significant difference between cases and controls (p=0.56)	Seasonal variation; inverse association with BMI	Early-life vitamin D levels are not associated with T1DM development
Habibian et al., [17] (2019)	Deficient/Insufficient vs sufficient (<30 vs >30 ng/ml)	Lower SCP/FCP in deficient; higher in sufficient	SCP was significantly higher in sufficient vitamin D (p<0.01); FCP was not significant	Vitamin D protects β-cells via anti-apoptotic, anti-inflammatory, Th1/Th2 modulation; VDR polymorphisms modulate effect	Adequate vitamin D supports β-cell function and insulin secretion; genetic factors influence the vitamin D effect in T1DM
The et al., [18] (2013)	Deficient/Inadequate vs sufficient (<50 vs ≥50 nmol/L)	Lower insulin sensitivity/ higher IR	Cross-sectional OR: 0.70 (0.57-0.85), β: 0.50; longitudinal OR: 0.69 (0.63-1.14), β: 0.12 (not significant)	Associated with higher BMI, higher triglycerides, and higher HbA1c	Higher vitamin D linked to lower IR cross-sectionally; longitudinal effect not significant

Risk of Bias Assessment

The risk of bias of the included studies was evaluated using appropriate tools based on study design. The controlled clinical trial by Moreira and colleagues [11] was assessed using the Cochrane RoB 2 tool and demonstrated low risk of bias across all five domains, including the randomisation process, deviations from intended interventions, missing outcome data, measurement of the outcome, and selection of the reported result, resulting in an overall low risk of bias judgement (Table 3).

**Table 3:** Risk of bias assessment using Cochrane Risk of Bias tool 2 (randomised controlled trial)

Author (year)	Randomisation process	Deviations from intended interventions	Missing outcome data	Measurement of outcome	Selection of reported results	Overall risk of bias
Moreira et al., [11] (2025)	Low risk	Low risk	Low risk	Low risk	Low risk	Low risk

Among the seven studies assessed using the adapted Newcastle-Ottawa Scale, five were rated as good quality [12,14,16-18], and two were rated as fair quality [13,15]. Kamiński and colleagues [12] and Savastio and colleagues [14] each received scores of 8/9, demonstrating adequate selection, good comparability with adjustment for important confounders, and robust outcome assessment. Mäkinen and colleagues [16] achieved the highest score of 9/9, reflecting its population-based nested case-control design, comprehensive confounder adjustment, and complete outcome data. Habibian and colleagues [17] scored 8/9, with strengths in selection and outcome assessment but partial confounder adjustment. The et al. [18] received 7/9, with limitations in selection due to potential sampling issues, but good comparability and outcome measurement. Tunc and colleagues [13] and Al Zahrani and Al Shaikh [15] were both rated as fair quality with scores of 6/9, primarily due to limited adjustment for confounding variables and, in the case of Al Zahrani and Al Shaikh [15], the absence of an explicitly defined vitamin D deficiency threshold and retrospective design. The complete risk of bias assessments are presented in Table 4.

**Table 4:** Risk of bias assessment using the adapted Newcastle-Ottawa Scale for cross-sectional and cohort studies

Author (Year)	Selection (maximum 4 stars)	Comparability (maximum 2 stars)	Outcome (maximum 3 stars)	Total score (maximum 9 stars)	Quality rating
Kamiński et al., [12] (2021)	★★★ (3)	★★ (2)	★★★ (3)	8/9	Good
Tunc et al., [13] (2011)	★★ (2)	★ (1)	★★★ (3)	6/9	Fair
Savastio et al., [14] (2016)	★★★ (3)	★★ (2)	★★★ (3)	8/9	Good
Al Zahrani and Al Shaikh, [15] (2019)	★★★ (3)	★ (1)	★★ (2)	6/9	Fair
Mäkinen et al., [16] (2016)	★★★★ (4)	★★ (2)	★★★ (3)	9/9	Good
Habibian et al., [17] (2019)	★★★ (3)	★★ (2)	★★★ (3)	8/9	Good
The et al., [18] (2013)	★★ (2)	★★ (2)	★★★ (3)	7/9	Good

Discussion

This systematic review synthesises evidence from eight studies examining the relationship between hypovitaminosis D and insulin resistance in type 1 diabetes mellitus, incorporating both clinical and mechanistic findings. The collective evidence presents a complex picture, with the majority of cross-sectional studies demonstrating significant associations between low vitamin D status and markers of insulin resistance, while interventional and longitudinal data yield more inconsistent results. These findings have important implications for understanding the potential role of vitamin D in metabolic control among individuals with type 1 diabetes and highlight critical areas for future research.

The prevalence of hypovitaminosis D among individuals with type 1 diabetes was consistently high across the included studies, though estimates varied considerably depending on the threshold definitions employed. Moreira and colleagues reported that 79% of Brazilian children and adolescents with type 1 diabetes had vitamin D deficiency defined as serum 25(OH)D below 30 ng/mL [11], while Kamiński and colleagues found that 47.3% of Polish adults were deficient below 20 ng/mL, with 14.5% exhibiting severe deficiency below 10 ng/mL [12]. This high prevalence aligns with existing literature documenting increased risk of vitamin D deficiency in autoimmune conditions generally, and in type 1 diabetes specifically [19]. Several factors may contribute to this phenomenon, including shared genetic predisposition affecting vitamin D metabolism, disease-related lifestyle modifications such as sun avoidance, and potential increased urinary loss of vitamin D binding protein in individuals with diabetic nephropathy [20]. The observation by Mäkinen et al. of seasonal variation in 25(OH)D levels [16] further supports the role of environmental factors in determining vitamin D status in this population, consistent with population-based studies demonstrating winter-time declines in vitamin D concentrations even in healthy individuals.

The association between vitamin D status and insulin resistance measures emerged as a consistent finding across most cross-sectional studies. Kamiński and colleagues demonstrated a positive correlation between 25(OH)D levels and estimated glucose disposal rate (Rs=0.27, p<0.01), with severe deficiency conferring a fourfold increased odds of insulin resistance (OR=4.19) [12]. This magnitude of effect is clinically significant and aligns with findings from large epidemiological studies in non-diabetic populations, such as the National Health and Nutrition Examination Survey, which reported inverse associations between vitamin D levels and homeostasis model assessment of insulin resistance [21]. Tunc and colleagues similarly found a significant negative correlation between 25(OH)D and insulin requirements (r=-0.215, p=0.032) [13], suggesting that vitamin D status may influence peripheral insulin sensitivity and thereby exogenous insulin needs. This relationship has been corroborated by studies in type 2 diabetes populations, where vitamin D repletion has been associated with improved insulin sensitivity indices [22]. The SEARCH Nutrition Ancillary Study by The et al. provided particularly robust evidence, demonstrating a significant cross-sectional association between higher vitamin D levels and lower insulin resistance (OR: 0.70, 95% CI: 0.57-0.85) in a large, well-characterised cohort of youth with type 1 diabetes [18].

However, not all studies supported this association. Al Zahrani and Al Shaikh found no relationship between vitamin D levels and HbA1c (R=0.04, p=0.60) in their Saudi Arabian cohort [15], a finding that may reflect the uniformly low vitamin D status in this population, limiting the ability to detect dose-response relationships, or the relatively crude nature of HbA1c as a proxy for insulin resistance. The discrepancy between cross-sectional and interventional findings in both our review and the broader literature suggests that while vitamin D deficiency may be a marker of metabolic dysregulation, it may not be a straightforward therapeutic target in all populations.

The mechanistic studies included in this review provide biological plausibility for the observed associations. Habibian and colleagues demonstrated that children with sufficient vitamin D levels had significantly higher stimulated C-peptide levels compared to those with deficiency (p<0.01) [17], suggesting that vitamin D may preserve residual beta-cell function through immunomodulatory mechanisms. This finding aligns with experimental studies showing that 1,25-dihydroxyvitamin D3 suppresses dendritic cell maturation and promotes regulatory T-cell differentiation, thereby attenuating autoimmune destruction of pancreatic beta-cells. The observation by Habibian and colleagues that vitamin D receptor gene polymorphisms modulated this effect [17] is particularly important, as it suggests genetic heterogeneity may influence individual responses to vitamin D status and supplementation. This pharmacogenetic aspect has been explored in other autoimmune conditions, with polymorphisms in the VDR gene associated with differential susceptibility to type 1 diabetes itself, and may explain some of the variability in clinical outcomes observed across studies.

Savastio and colleagues proposed that hypovitaminosis D worsens metabolic control through both reduced insulin sensitivity and direct effects on peripheral tissue function [14], a mechanism supported by studies demonstrating vitamin D receptors on skeletal muscle and adipose tissue. Vitamin D is known to regulate insulin receptor expression and enhance insulin signal transduction through effects on peroxisome proliferator-activated receptor delta and other downstream targets [23].

The effects of vitamin D supplementation on metabolic outcomes in type 1 diabetes were examined in two studies with conflicting results. Moreira and colleagues found that while supplementation effectively corrected deficiency, increasing 25(OH)D from 19.2 to 30.9 ng/mL (p<0.001), this did not translate into significant improvement in glycaemic control as measured by HbA1c [11]. In contrast, Savastio and colleagues reported that supplementation improved 25(OH)D status and was associated with enhanced insulin sensitivity and better glycaemic outcomes [14]. These divergent findings mirror the broader literature on vitamin D supplementation in diabetes. A meta-analysis by Wu and colleagues of randomised controlled trials in type 2 diabetes found that vitamin D supplementation significantly improved glycaemic control only in studies with baseline vitamin D deficiency and adequate intervention duration [24]. Similarly, the Vitamin D and Type 2 Diabetes (D2d) study, a large multicentre randomised controlled trial, reported that vitamin D supplementation reduced the risk of progression from prediabetes to diabetes, though the effect was modest and limited to those with baseline vitamin D deficiency [25]. The lack of effect in the Moreira study may reflect the relatively short intervention duration (12 weeks), which may be insufficient to observe changes in HbA1c given the erythrocyte lifespan of approximately 120 days, or the use of HbA1c rather than more sensitive measures of insulin resistance such as euglycaemic clamp studies [11].

Longitudinal evidence on the vitamin D-insulin resistance relationship was limited but noteworthy. The et al. found no significant association between vitamin D status and insulin resistance over time in their longitudinal analysis of the SEARCH cohort (OR: 0.69, 95% CI: 0.63-1.14) [18], despite a significant cross-sectional association. This finding suggests that vitamin D may be a marker of prevailing metabolic health rather than a predictor of future insulin resistance, or that single-timepoint measurements of 25(OH)D may not adequately capture long-term vitamin D exposure relevant to metabolic outcomes. Mäkinen and colleagues found no difference in 25(OH)D concentrations between children who progressed to type 1 diabetes and controls (p=0.56) [16], suggesting that vitamin D status in early life may not be a primary determinant of autoimmune diabetes development.

The heterogeneity in findings across studies likely reflects multiple factors. First, the use of different thresholds for defining vitamin D deficiency complicates cross-study comparisons, as illustrated by the range of definitions from <10 ng/mL to <30 ng/mL across included studies. This variability in cut-points reflects ongoing debate in the field regarding optimal vitamin D status for metabolic health [26]. Second, studies employed diverse measures of insulin resistance, including eGDR, insulin dose requirements, HbA1c, and insulin sensitivity scores, which capture different aspects of insulin action and are not directly comparable. The eGDR, while validated in type 1 diabetes populations, incorporates HbA1c, blood pressure, and waist circumference, and may reflect broader metabolic health rather than isolated insulin resistance. Third, the age ranges of study populations varied considerably, from young children to adults, and insulin resistance manifests differently across the lifespan, with puberty representing a period of physiological insulin resistance that may interact with vitamin D status. Fourth, geographic variation in sun exposure, dietary vitamin D intake, and genetic background may influence both baseline vitamin D status and the relationship between vitamin D and metabolic outcomes [27].

Several methodological strengths of the included studies enhance confidence in the synthesised findings. The majority of studies (six out of eight) were assessed as either low risk of bias or good quality, with robust vitamin D measurement methods including chemiluminescence immunoassay (CLIA), enzyme-linked immunosorbent assay (ELISA), high-performance liquid chromatography (HPLC), and chemiluminescence, all of which are validated for 25(OH)D quantification. The inclusion of large cohorts such as the SEARCH study [18] and population-based nested case-control designs [16] provides generalisable estimates. The mechanistic studies incorporating VDR genotyping [11,17] represent methodologically rigorous approaches to understanding biological pathways. However, important limitations must be acknowledged when interpreting the evidence base. The predominance of cross-sectional designs precludes causal inference, and the limited number of intervention trials restricts conclusions regarding therapeutic efficacy. The variability in outcome measures and deficiency thresholds, as previously discussed, limits the feasibility of meta-analysis and introduces heterogeneity that must be considered qualitatively.

Limitations

This systematic review has several limitations that should be considered when interpreting the findings. First, the number of included studies was relatively small (n=8), limiting the robustness of synthesised conclusions and precluding quantitative meta-analysis. Second, the heterogeneity in study designs, populations, vitamin D assessment methods, deficiency thresholds, and outcome measures complicated direct comparisons across studies and may account for some of the observed variability in findings. Third, the predominance of cross-sectional studies limits the ability to infer causality or direction of effect, as reverse causation cannot be excluded, whereby insulin resistance or its metabolic consequences might influence vitamin D status through effects on behaviour, metabolism, or vitamin D binding protein concentrations. Fourth, publication bias cannot be ruled out, as studies with null findings may be less likely to be published, potentially overestimating the strength of associations. Fifth, the inclusion of studies published in English only may have introduced language bias. Sixth, several included studies had limited adjustment for potential confounders such as physical activity, sun exposure, dietary intake, and seasonal variation, which may have influenced observed associations. Seventh, the generalisability of findings may be limited by the geographic distribution of studies, with underrepresentation of certain regions and ethnic groups. Finally, the lack of standardised protocols for vitamin D supplementation across intervention studies limits conclusions regarding optimal dosing, duration, and therapeutic thresholds for improving insulin resistance in type 1 diabetes.

## Conclusions

Hypovitaminosis D is highly prevalent among individuals with type 1 diabetes and is associated with insulin resistance in the majority of cross-sectional studies. Mechanistic evidence supports biological plausibility through effects on beta-cell function, inflammatory pathways, VDR polymorphisms, and broader metabolic parameters. However, interventional and longitudinal evidence remains limited and inconsistent, with supplementation trials showing conflicting results and prospective studies failing to demonstrate significant associations over time. The findings suggest that while vitamin D deficiency may serve as a marker of metabolic dysregulation in type 1 diabetes, its role as a modifiable risk factor for insulin resistance requires further investigation through well-designed randomised controlled trials with adequate duration, standardised protocols, and sensitive measures of insulin resistance. Future research should prioritise identifying patient subgroups most likely to benefit from supplementation based on baseline vitamin D status, genetic background, and clinical characteristics, and should incorporate mechanistic outcomes to elucidate pathways linking vitamin D to insulin sensitivity. Until such evidence is available, optimisation of vitamin D status in individuals with type 1 diabetes remains clinically prudent for bone health and general wellbeing, but cannot be definitively recommended as a strategy for improving insulin resistance or glycaemic control.

## Appendices

**Table 5:** Search strategy for each database

Database	Comprehensive search string
PubMed^®^	("Type 1 Diabetes Mellitus"[MeSH] OR "T1DM" OR "Type 1 Diabetes" OR "Juvenile Diabetes") AND ("Vitamin D Deficiency"[MeSH] OR "Hypovitaminosis D" OR "Vitamin D insufficiency" OR "25-hydroxyvitamin D" OR "25(OH)D") AND ("Insulin Resistance"[MeSH] OR "IR" OR "Insulin Sensitivity" OR "HOMA-IR" OR "glucose disposal rate" OR "eGDR")
BMJ Journals	("Type 1 Diabetes" OR "T1DM" OR "Juvenile Diabetes") AND ("Vitamin D deficiency" OR "Hypovitaminosis D" OR "Vitamin D insufficiency" OR "25-hydroxyvitamin D" OR "25(OH)D") AND ("Insulin Resistance" OR "Insulin Sensitivity" OR "HOMA-IR" OR "glucose disposal rate" OR "eGDR")
Scopus^®^	TITLE-ABS-KEY("Type 1 Diabetes Mellitus" OR "T1DM" OR "Juvenile Diabetes") AND TITLE-ABS-KEY("Vitamin D deficiency" OR "Hypovitaminosis D" OR "Vitamin D insufficiency" OR "25-hydroxyvitamin D" OR "25(OH)D") AND TITLE-ABS-KEY("Insulin Resistance" OR "Insulin Sensitivity" OR "HOMA-IR" OR "glucose disposal rate" OR "eGDR")
IEEE Xplore^®^	("Type 1 Diabetes" OR "T1DM" OR "Juvenile Diabetes") AND ("Vitamin D deficiency" OR "Hypovitaminosis D" OR "Vitamin D insufficiency" OR "25-hydroxyvitamin D" OR "25(OH)D") AND ("Insulin Resistance" OR "Insulin Sensitivity" OR "HOMA-IR" OR "glucose disposal rate" OR "eGDR")
Web of Science™	TS=("Type 1 Diabetes Mellitus" OR "T1DM" OR "Juvenile Diabetes") AND TS=("Vitamin D deficiency" OR "Hypovitaminosis D" OR "Vitamin D insufficiency" OR "25-hydroxyvitamin D" OR "25(OH)D") AND TS=("Insulin Resistance" OR "Insulin Sensitivity" OR "HOMA-IR" OR "glucose disposal rate" OR "eGDR")
